# Tmem119-EGFP and Tmem119-CreERT2 Transgenic Mice for Labeling and Manipulating Microglia

**DOI:** 10.1523/ENEURO.0448-18.2019

**Published:** 2019-08-26

**Authors:** Tobias Kaiser, Guoping Feng

**Affiliations:** 1 McGovern Institute for Brain Research, Massachusetts Institute of Technology, Cambridge, Massachusetts 02139; 2 Department of Brain and Cognitive Sciences, Massachusetts Institute of Technology, Cambridge, Massachusetts 02139; 3 Stanley Center for Psychiatric Research, Broad Institute of MIT and Harvard, Cambridge, Massachusetts 02142

**Keywords:** CreERT2, EGFP, macrophage, microglia, Tmem119, transgenic

## Abstract

Microglia are specialized brain-resident macrophages with important functions in health and disease. To improve our understanding of these cells, the research community needs genetic tools to identify and control them in a manner that distinguishes them from closely related cell types. We have targeted the recently discovered microglia-specific *Tmem119* gene to generate knock-in mice expressing EGFP (JAX#031823) or CreERT2 (JAX#031820) for the identification and manipulation of microglia, respectively. Genetic characterization of the locus and qPCR-based analysis demonstrate correct positioning of the transgenes and intact expression of endogenous *Tmem119* in the knock-in mouse models. Immunofluorescence analysis further shows that parenchymal microglia, but not other brain macrophages, are completely and faithfully labeled in the EGFP-line at different time points of development. Flow cytometry indicates highly selective expression of EGFP in CD11b^+^CD45lo microglia. Similarly, immunofluorescence and flow cytometry analyses using a Cre-dependent reporter mouse line demonstrate activity of CreERT2 primarily in microglia upon tamoxifen administration with the caveat of activity in leptomeningeal cells. Finally, flow cytometric analyses reveal absence of EGFP expression and minimal activity of CreERT2 in blood monocytes of the *Tmem119-EGFP* and *Tmem119-CreERT2* lines, respectively. These new transgenic lines extend the microglia toolbox by providing the currently most specific genetic labeling and control over these cells in the myeloid compartment of mice.

## Visual Abstract

**Figure:**
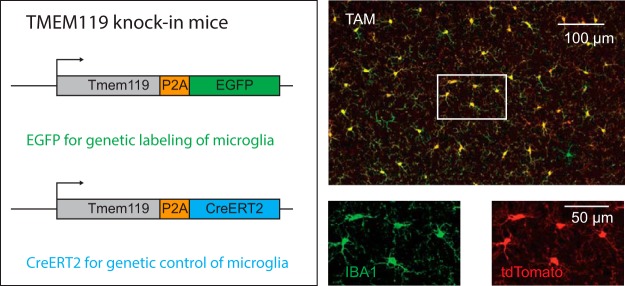

## Significance Statement

Tools that specifically label and manipulate only microglia are currently unavailable, but are critically needed to further our understanding of this cell type. Complementing and significantly extending recently introduced microglia-specific immunostaining methods that have quickly become a new standard in the field, we generated two mouse lines that label and control gene expression in microglia with high specificity and made them publicly available. Using these readily accessible mice, the research community will be able to study microglia biology with improved specificity.

## Introduction

Microglia are specialized brain-resident macrophages that comprise 5–12% of the glial cells in the adult mouse brain (Lawson et al., 1990). Under physiologic conditions, phagocytic and secretory activity of these glia support neurogenesis, development of neuronal connectivity, and survival of neurons (Stevens et al., 2007; Sierra et al., 2010; Paolicelli et al., 2011; Schafer et al., 2012; Ueno et al., 2013; Weinhard et al., 2018). Complementing these homeostatic functions, microglia react to perturbations, which has been shown in the context of vascular injury, multiple sclerosis lesions, and neurodegeneration (Itagaki et al., 1989; Davalos et al., 2005; Ransohoff, 2016; Aguzzi and Zhu, 2017; Keren-Shaul et al., 2017; Mathys et al., 2017; O’Loughlin et al., 2018). In the future, many of these processes and their potential for therapeutic targeting will be further examined, and understanding of both homeostatic and disease-related contributions of microglia will critically depend on means to specifically identify and control them. Several mouse lines are currently available for either fluorescent labeling or Cre-expression that harness the loci of putative microglia signature genes *Tie2*, *Runx1*, *Csf1r*, *Aif1*, *Lyz2*, *Itgam*, *Sall1*, or *CX3cr1* (Clausen et al., 1999; Sasmono et al., 2003; Ferron and Vacher, 2005; Hirasawa et al., 2005; Samokhvalov et al., 2007; Ginhoux et al., 2010; Parkhurst et al., 2013; Yona et al., 2013; Buttgereit et al., 2016). Collectively, these mouse lines have been instrumental in gaining insights on microglia. However, furthering our understanding of microglia using the currently available lines is complicated by the fact that it is difficult to distinguish microglia from other closely related peripheral and central cell types such as blood monocytes as well as perivascular, choroid plexus, and meningeal macrophages (Wieghofer and Prinz, 2016; Haimon et al., 2018).

Recent advances in RNA sequencing and other cell profiling technologies have enabled the discovery of cell-type-specific signature genes (Butovsky et al., 2014). Among these, transmembrane protein 119 (*Tmem119*) is highly and exclusively expressed in microglia in the brains of mice and humans (Bennett et al., 2016; Satoh et al., 2016). Although TMEM119-specific antibodies are already widely used as a gold standard for immunohistochemical methods, faithful fluorescent reporter lines and inducible Cre-lines that allow *in vivo* observation and manipulation are currently not available. Here, we report the generation and characterization of knock-in mouse lines *Tmem119-EGFP* (JAX#031823) and *Tmem119-CreERT2* (JAX#031820), where microglia express EGFP and CreERT2, respectively, while preserving endogenous *Tmem119* expression. We demonstrate that EGFP is expressed throughout the brain and that the tag is confined to microglia only, without significantly labeling other brain macrophages. We further provide evidence that the inducible Cre is primarily active in microglia by crossing to the conditional fluorescent reporter mouse line Ai14. In these mice, we also observe activity in leptomeningeal cells that line the surface of the brain and penetrate deep into the brain ensheathing some large blood vessels. Finally, we demonstrate minimal to absent transgene expression in monocytes of the mice. These publicly available mouse lines provide valuable tools for the functional study of bona fide microglia.

## Materials and Methods

### Animal work

All animal procedures were performed in accordance with MIT’s animal care regulations. Overall, >30 animals of >10 litters were generated per line and mice were crossed to C57BL/6J up to generation N3. For all experiments, at least three independent mice were analyzed, which included both sexes and no apparent sex differences were observed.

### Generation of transgenic animals using CRISPR/Cas9

To generate *Tmem119* knock-in mice, donor DNA templates encoding ribosome-skipping peptide *porcine teschovirus-1 polyprotein* and *EGFP* (*P2A-EGFP*) or *CreERT2* (*P2A-CreERT2*) were synthesized. These sequences were flanked by sequences of 55–300 bp for EGFP and 1.5 kb for CreERT2 homologous to 5′ and 3′ regions around the *Tmem119* stop codon. These templates were injected into fertilized mouse oocytes together with a single crRNA (AGUCUCCCCCAGUGUCUAAC, Synthego) that cuts at the stop codon.

Donor DNA templates were generated by digesting pAAV-P2A-EGFP (sequence below) with XbaI and EcoRI (New England Biolabs) and inserting three gblocks (LHA, P2A-EGFP or P2A-CreERT2, RHA, purchased from IDT, sequences below) using Gibson cloning (HIFI assembly mix, New England Biolabs) according to the manufacturers’ protocols. Resulting plasmids were purified and sequenced. For CreERT2, the highly purified dsDNA plasmid was directly used as donor DNA in injections. For EGFP, single strand DNA (ssDNA) was produced using PCR with forward primers for left homology arms between 55 and 300 bp (5′-A*G*C*AACTGGTCCTCCTGAAA-3′ and 5′-CAAAGCCTGTGAAGGGTGGG-3′, respectively; * denoting phosphorothioate) and a reverse primer for a 55 bp right homology arm (5′-CAAAGAGGTGACCCTCAAGG-3′, with 5′ phosphorylation for lambda digest of antisense strand). Using these primers, large scale PCR with Takara PrimeStar was performed to obtain 40–60 μg of product. The PCR product was highly purified with the Qiagen PCR purification kit and subject to digestion of the antisense strand. Lambda exonuclease (New England Biolabs) was used to digest 20 μg of dsDNA at 37°C for 60 min. Complete digestion of dsDNA was confirmed by agarose gel electrophoresis and Sanger sequencing with sense- and antisense-binding primers.

Mixtures for injection of zygotes were prepared freshly on the morning of the day of injection. Briefly, water to a final volume of 100 μl was mixed with final concentrations of 10 mm TrisHcl buffer, 0.61 μm crRNA, 0.61 μm tracrRNA (sequence proprietary to Synthego) and heated to 95°C for 5 min. Heated mixtures were cooled to room temperature for 10 min and 30 ng/μl EnGen Cas9 protein (New England Biolabs) was added. Mixtures were incubated at 37°C for 15 min to form Cas9 to crRNA-tracrRNA complexes. Final concentrations of 5 ng/μl donor DNA (P2A-EGFP ssDNA or P2A-CreERT2 plasmid dsDNA) and 10 ng/μl recombinant RAD51 protein were added. Mixtures were kept on ice until use, when they were incubated at 37°C for 15 min followed by centrifugation at 10,000 rpm for 1 min to prevent clogging of the micropipette.

Injection of mixtures was conducted using standardized protocols of the transgenics facility in zygotes obtained from pure C57BL/B6N mice (Taconic).

### Genetic analysis and genotyping of Tmem119 and Ai14 mice

**Figure 1.**
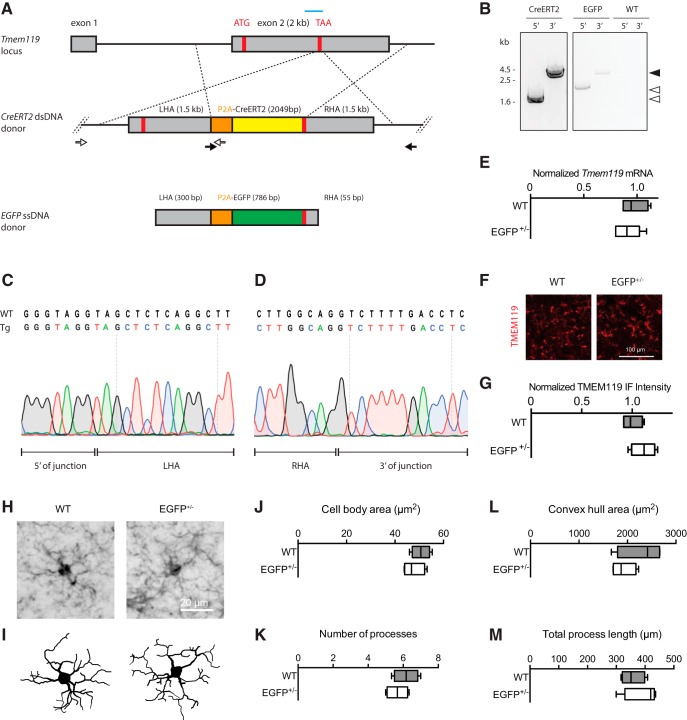
Generation and validation of *Tmem119-EGFP and Tmem119-CreERT2* knock-in lines. ***A***, Experimental strategy using a guide RNA (blue bar) to introduce a double-strand break at the *Tmem119* stop codon in mouse zygotes and an injection mixture containing *EGFP* ssDNA template with short homology arms of 55–300 bp or *CreERT2* dsDNA template with long 1.5 kb homology arms. Each knock-in is designed to cause in-frame insertion of P2A peptide followed by *EGFP* or *CreERT2* and a stop codon. Not drawn to scale. ***B***, Confirmation of insertion by amplification of fragments spanning the 5′ and 3′ junctions using sets of primers binding inside the template and outside the homology arms (***A***, open and closed arrows with arrowheads indicating corresponding products). ***C***, ***D***, Sanger sequencing across the 5′ and 3′ junction of the CreERT2 line and comparison to WT sequence indicate seamless insertion in the transgenic mice (Tg). ***E***, qPCR analysis of gene expression for *Tmem119* in WT controls and *Tmem119-EGFP*^+/−^ knock-in animals. *N* = 4 WT, *N* = 6 *Tmem119-EGFP*^+/−^ mice. ***F***, Representative TMEM119 immunoreactivity in WT controls and *Tmem119-EGFP*^+/−^ knock-in animals. ***G***, Relative intensity of TMEM119 immunostaining signal. *N* = 4 WT, *N* = 4 *Tmem119-EGFP*^+/−^ mice. ***H***, ***I***, Representative IBA-immunostained microglia and corresponding Neurolucida traces in WT and *Tmem119-EGFP*^+/−^ knock-in mice. ***J*–*M***, Quantified cell body area, number of processes, convex hull area, and total process length of microglia in WT and *Tmem119-EGFP*^+/−^ knock-in mice. *N* = 12 microglia from four mice per genotype. TAA (red bar), stop codon.

Founder mice were genetically examined by amplifying sequences spanning the 5′ and 3′ junction and including the entire inserted transgene. Specifically, high-quality DNA was obtained from ear punches using a tissue DNA extraction kit according to the manufacturer’s instructions (Macherey-Nagel). One amplicon spanning ∼1.5 kb (Fig. 1, open arrows) of these sequences was generated using Q5 polymerase with primers 5′ of LHA-F (GCCTCTGTCACTTAAGTTGG) and P2A-R (GCTTCAGCAGGCTGAAGTTA). A second amplicon of ∼ 2.5–3.5 kb (Fig. 1, closed arrows) was generated using primers Tmem-LHA-P2A-F (CAGTGTCGGAAGCGGAGCTA) and 3′ofRHA-R (GAAAGAGGAAGCTAGAAGGG). Both amplicons were purified and then sequenced using primers spanning the entire length using Sanger sequencing (GENEWIZ). Trace files were aligned to *in silico* assemblies and analyzed using Snapgene.

To genotype mice, ear tissue was prepared using an alkaline buffer (25 mm NaOH, 0.2 mm Na_2_EDTA, pH 12) at 95°C for 30 min. The lysed tissue solution was neutralized using an acidic buffer (40 mM TrisHcl, pH 5). PCR using primers WT-F GTCAGGAGGAGGCCCAGGAA, EGFP-F CTGCTGCCCGACAACCACTA, CreERT2-F ACCGCCTACATGCGCCCACT, and Common-R GTTTCCTGGGGTGCACCAGA yielded products of 400 bp for the wild-type allele and 320 bp for the EGFP or CreERT2 allele.

Ai14(RCL-tdT) mice were obtained from JAX (stock 007914) and maintained in house. Homozygous Ai14 mice were bred to heterozygous *Tmem119-CreERT2* mice to generate offspring for the analysis. Ai14 mice were genotyped according to the protocol provided by JAX.

### RNA isolation and quantitative PCR

Brain hemispheres from adult *Tmem119-EGFP*^+/−^ mice were dissected and snap-frozen in liquid nitrogen. Highly pure RNA was isolated using an RNeasy purification kit (Qiagen) following the manufacturer’s instructions. Equal amounts of RNA (1.5 μg per sample) were reverse transcribed using an iScript Advanced cDNA synthesis kit (Bio-Rad). Resulting cDNA was diluted 1:50 in ultrapure water. Quantitative PCR (qPCR) was conducted using SsOAdvanced Universal SYBR Green Supermix (Bio-Rad) on a CFX96 real-time system. Primers used were exon-spanning whenever possible and of the following sequences: Tmem119-F CCTTCACCCAGAGCTGGTTC, Tmem119-R GGCTACATCCTCCAGGAAGG, GAPDH-F GCCTTCCGTGTTCCTACC, GAPDH-R CCTCAGTGTAGCCCAAGATG, b-actin-F CTAAGGCCAACCGTGAAAAG, b-actin-R ACCAGAGGCATACAGGGACA. Differential gene expression analysis was performed using built-in software for the Bio-Rad CFX96 real-time system.

### Tamoxifen administration

Tamoxifen (Sigma-Aldrich, T5648) was dissolved in corn oil at 20 mg/ml under agitation for several hours in the dark and kept at room temperature for 2–3 d. Using needles from Harvard Apparatus (52-4025), separately housed adult animals were fed via oral gavage for 3 d. Needles were rinsed between days and syringes were replaced. At time of administration, the mice weighed ∼19–26 g and received 250–400 μl of the 20 mg/ml tamoxifen solution, corresponding to 0.2 mg/g body weight. Postnatal day (P)2 mice weighing ∼2 g received 5 μl of the 20 mg/ml solution, corresponding to 50 μg/g body weight per day for 3 consecutive days. The health of the mice was monitored daily. At least 7 d week after the last dose, mice were killed by transcardiac perfusion.

### Immunofluorescence staining and imaging

Adult mice were deeply anesthetized and perfused with 25 ml PBS followed by 25 ml 4% paraformaldehyde (PFA) in PBS. Early postnatal mice (P3) were perfused with 8 ml PBS and 8 ml PFA. Brains were surgically removed and postfixed in the fixative at 4°C for 24 h. Fixed brains were washed once in PBS and sliced into 100-μm-thick sagittal slices using a Leica VT1000S. Slices were washed twice in PBS, permeabilized in 1.2% TX100 in PBS for 15 min, washed twice in PBS, and subject to incubation in blocking solution (5% normal goat serum, 2% bovine serum albumin, 0.2% TX100 in PBS). Blocked sections were incubated with primary antibodies for IBA1 (1:500; Synaptic Systems, 234006), GFP (1:1000, Invitrogen, A11122; 1:500, Aves Labs, GFP-1020), TMEM119 (1:1000; Abcam, ab209064), Olig2 (1:1000; Millipore, AB9610), NeuN (1:1000; Millipore, MAB377), GFAP (1:1000; Sigma-Aldrich, G9269), or CD163 (1:200; Abcam, ab182422, antigen retrieval required) for 24 h at 4°C. Primary antibody incubation was followed by three washes in PBS and incubation with species-matched and Alexa fluorophore-conjugated secondary antibodies raised in goat (Invitrogen; 1:1000) for 2 h. DAPI (1:10.000) was included in a washing step or secondary antibody incubation. Slices were washed three times in PBS and mounted and coverslipped using VECTASHIELD H-1000 mounting medium.

For immunostaining of CD163, an antigen retrieval step was included. Briefly, vibratome slices were washed twice in PBS, incubated in a retrieval buffer (10 mm Sodium citrate, pH 8.5) for 5 min, followed by incubation in the retrieval buffer at 80°C for 30 min. Sections were cooled to room temperature and washed twice in PBS. Blocking and immunostaining were then conducted as described for other antibodies in the immunofluorescence staining and imaging methods section.

For imaging, slides were scanned on an Olympus FluoView FV1000 fixed stage confocal microscope (high-power magnifications) or Olympus BX61 epifluorescence microscope (sagittal section montage) using built-in software. For coexpression analysis in the *Tmem119-EGFP* line, maximum projections of four *z*-stacks from three 20× high-power fields (∼100 cells per region) were counted manually.

For microglia morphology analysis, *z*-stacks were acquired at 20× magnification with 2× digital zoom and 1.2 μm step size. Images were imported as stacks into Neurolucida software. Cell bodies in the middle of the stacks with complete process arbors were selected and manually traced. Raw trace files were imported into Neurolucida explorer software and convex hull area, number of processes and total process length were determined using the convex hull analysis and branched structure analysis functions. Rendered traces were exported as monochrome vector graphics. Cell body areas were separately determined in FIJI using the polygon tool.

### Cell suspension preparation

Microglia and blood monocytes for single-cell suspensions for flow cytometry were prepared as follows. Mice were deeply anesthetized with isoflurane and transcardially perfused with ice-cold HBSS. During perfusion, 2 ml of whole blood was collected from the right atrium into tubes containing 40 μl of 10% (w/v) EDTA. The 2 ml of whole blood were added to 40 ml of red blood cell lysis buffer (Abcam, ab204733) and incubated for 10 min at room temperature to lyse red blood cells. The suspension containing lysed RBCs and white blood cells was spun down at 300 × *g* for 5 min at 4°C, resuspended in 10 ml ice-cold HBSS, and pelleted again at 300 × *g* for 5 min at 4°C. WBCs were resuspended in 500 μl ice-cold FACS buffer (HBSS, 0.5% BSA, 1 mm EDTA) and subject to staining. In parallel with the WBC enrichment, brains were rapidly dissected into 2 ml ice-cold HBSS and cerebella and brainstem were removed. Brains were minced into small pieces and transferred to a Dounce homogenizer containing 5 ml ice-cold HBSS with 20 μg/ml DNase I (Worthington, DPRF, LS006343). Tissue chunks were homogenized with 15 loose and 15 more tight strokes and the homogenate was transferred to a 50 ml falcon tube through a pre-wet 70 μm strainer. The strainer was rinsed with HBSS to top off the volume of each sample to 10 ml. Filtered homogenates were transferred to 15 ml falcon tubes and spun at 300 × *g* for 5 min at 4°C. Supernatants were carefully removed, and pellets resuspended in 10 ml of ice-cold 40% Percoll in HBSS. Samples were spun at 500 × *g* for 30 min at 4°C with full acceleration and deceleration. Myelin and debris from the supernatant were carefully removed and pellets resuspended in 10 ml ice-cold HBSS. Following another spin at 300 × *g* for 5 min at 4°C, the supernatant was removed and the microglial pellet resuspended in 1 ml FACS buffer (0.5% BSA HBSS, 1 mm EDTA).

### Staining for flow cytometry

Suspensions of white blood cells and microglia were transferred to 2 ml Eppendorf microcentrifuge tubes, and a small fraction of sample was removed for single-color controls. To stain dead cells, live/dead violet (1:500–1:1000; ThermoFisher, L34955) was added and incubated for 5 min on ice. Tubes with live/dead-stained cells were topped off to 2 ml with FACS buffer (0.5% BSA HBSS, 1 mm EDTA) and spun down at 300 × *g* for 5 min at 4°C. Pellets were resuspended and incubated with 1:200 Mouse Fc Block (BD, 2.4G2, 553142) on ice for 15 min. Samples were incubated with 1:200 rat anti CD45-APC/Cy7 (BioLegend, 103115) and rat anti CD11b-PE/Cy5 (BioLegend, 101209) at 4°C. Tubes were topped off to 2 ml with ice-cold FACS buffer and microglia pelleted at 300 × *g* for 5 min at 4°C. Supernatants were removed and microglia resuspended in 500 μl. Resuspended microglia were filtered through corning strainer polystyrene tubes (Corning, 352235). Flow cytometry data were acquired on Aria II, Fortessa HTS, and LSRII HTS, and analyzed using FlowJo.

### Statistical analysis

Quantitative data from the qPCR, immunofluorescence, and microglia morphology experiments were analyzed using GraphPad prism. Testing for normality was not possible because *N* = 4–6 (4–6 independent mice were used per group). Not assuming Gaussian distribution of the data, a nonparametric test (Mann–Whitney) was used to compare median expression in both groups.

### DNA sequences

*pAAV-P2A-EGFP*

CCTGCAGGCAGCTGCGCGCTCGCTCGCTCACTGAGGCCGCCCGGGCAAAGCCCGGGCGTCGGGCGACCTTTGGTCGCCCGGCCTCAGTGAGCGAGCGAGCGCGCAGAGAGGGAGTGGCCAACTCCATCACTAGGGGTTCCTGCGGCCGCACGCGTTTAATTAAGTGTCTAGACTGCAGAGGGCCCTGCGTATGAGTGCAAGTGGGTTTTAGGACCAGGATGAGGCGGGGTGGGGGTGCCTACCTGACGACCGACCCCGACCCACTGGACAAGCACCCAACCCCCATTCCCCAAATTGCGCATCCCCTATCAGAGAGGGGGAGGGGAAACAGGATGCGGCGAGGCGCGTGCGCACTGCCAGCTTCAGCACCGCGGACAGTGCCTTCGCCCCCGCCTGGCGGCGCGCGCCACCGCCGCCTCAGCACTGAAGGCGCGCTGACGTCACTCGCCGGTCCCCCGCAAACTCCCCTTCCCGGCCACCTTGGTCGCGTCCGCGCCGCCGCCGGCCCAGCCGGACCGCACCACGCGAGGCGCGAGATAGGGGGGCACGGGCGCGACCATCTGCGCTGCGGCGCCGGCGACTCAGCGCTGCCTCAGTCTGCGGTGGGCAGCGGAGGAGTCGTGTCGTGCCTGAGAGCGCAGTCGAGAAACCGGCTAGAGGATCCTTCGAAACCGGTGCTAGCAGCGCTGTTAACGGAAGCGGAGCCACTAACTTCTCCCTGTTGAAACAAGCAGGGGATGTCGAAGAGAATCCCGGGCCAATGGTGAGCAAGGGCGAGGAGCTGTTCACCGGGGTGGTGCCCATCCTGGTCGAGCTGGACGGCGACGTAAACGGCCACAAGTTCAGCGTGTCCGGCGAGGGCGAGGGCGATGCCACCTACGGCAAGCTGACCCTGAAGTTCATCTGCACCACCGGCAAGCTGCCCGTGCCCTGGCCCACCCTCGTGACCACCTTCGGCTACGGCCTGCAGTGCTTCGCCCGCTACCCCGACCACATGAAGCAGCACGACTTCTTCAAGTCCGCCATGCCCGAAGGCTACGTCCAGGAGCGCACCATCTTCTTCAAGGACGACGGCAACTACAAGACCCGCGCCGAGGTGAAGTTCGAGGGCGACACCCTGGTGAACCGCATCGAGCTGAAGGGCATCGACTTCAAGGAGGACGGCAACATCCTGGGGCACAAGCTGGAGTACAACTACAACAGCCACAACGTCTATATCATGGCCGACAAGCAGAAGAACGGCATCAAGGTGAACTTCAAGATCCGCCACAACATCGAGGACGGCAGCGTGCAGCTCGCCGACCACTACCAGCAGAACACCCCCATCGGCGACGGCCCCGTGCTGCTGCCCGACAACCACTACCTGAGCTACCAGTCCGCCCTGAGCAAAGACCCCAACGAGAAGCGCGATCACATGGTCCTGCTGGAGTTCGTGACCGCCGCCGGGATCACTCTCGGCATGGACGAGCTGTACAAGTAAGGCGCGCCCCTGCAGGGAATTCGATATCAAGCTTATCGATAATCAACCTCTGGATTACAAAATTTGTGAAAGATTGACTGGTATTCTTAACTATGTTGCTCCTTTTACGCTATGTGGATACGCTGCTTTAATGCCTTTGTATCATGCTATTGCTTCCCGTATGGCTTTCATTTTCTCCTCCTTGTATAAATCCTGGTTGCTGTCTCTTTATGAGGAGTTGTGGCCCGTTGTCAGGCAACGTGGCGTGGTGTGCACTGTGTTTGCTGACGCAACCCCCACTGGTTGGGGCATTGCCACCACCTGTCAGCTCCTTTCCGGGACTTTCGCTTTCCCCCTCCCTATTGCCACGGCGGAACTCATCGCCGCCTGCCTTGCCCGCTGCTGGACAGGGGCTCGGCTGTTGGGCACTGACAATTCCGTGGTGTTGTCGGGGAAATCATCGTCCTTTCCTTGGCTGCTCGCCTATGTTGCCACCTGGATTCTGCGCGGGACGTCCTTCTGCTACGTCCCTTCGGCCCTCAATCCAGCGGACCTTCCTTCCCGCGGCCTGCTGCCGGCTCTGCGGCCTCTTCCGCGTCTTCGCCTTCGCCCTCAGACGAGTCGGATCTCCCTTTGGGCCGCCTCCCCGCATCGATACCGAGCGCTGCTCGAGAGATCTACGGGTGGCATCCCTGTGACCCCTCCCCAGTGCCTCTCCTGGCCCTGGAAGTTGCCACTCCAGTGCCCACCAGCCTTGTCCTAATAAAATTAAGTTGCATCATTTTGTCTGACTAGGTGTCCTTCTATAATATTATGGGGTGGAGGGGGGTGGTATGGAGCAAGGGGCAAGTTGGGAAGACAACCTGTAGGGCCTGCGGGGTCTATTGGGAACCAAGCTGGAGTGCAGTGGCACAATCTTGGCTCACTGCAATCTCCGCCTCCTGGGTTCAAGCGATTCTCCTGCCTCAGCCTCCCGAGTTGTTGGGATTCCAGGCATGCATGACCAGGCTCAGCTAATTTTTGTTTTTTTGGTAGAGACGGGGTTTCACCATATTGGCCAGGCTGGTCTCCAACTCCTAATCTCAGGTGATCTACCCACCTTGGCCTCCCAAATTGCTGGGATTACAGGCGTGAACCACTGCTCCCTTCCCTGTCCTTCTGATTTTGTAGGTAACCACGTGCGGACCGAGCGGCCGCAGGAACCCCTAGTGATGGAGTTGGCCACTCCCTCTCTGCGCGCTCGCTCGCTCACTGAGGCCGGGCGACCAAAGGTCGCCCGACGCCCGGGCTTTGCCCGGGCGGCCTCAGTGAGCGAGCGAGCGCGCAGCTGCCTGCAGGGGCGCCTGATGCGGTATTTTCTCCTTACGCATCTGTGCGGTATTTCACACCGCATACGTCAAAGCAACCATAGTACGCGCCCTGTAGCGGCGCATTAAGCGCGGCGGGTGTGGTGGTTACGCGCAGCGTGACCGCTACACTTGCCAGCGCCCTAGCGCCCGCTCCTTTCGCTTTCTTCCCTTCCTTTCTCGCCACGTTCGCCGGCTTTCCCCGTCAAGCTCTAAATCGGGGGCTCCCTTTAGGGTTCCGATTTAGTGCTTTACGGCACCTCGACCCCAAAAAACTTGATTTGGGTGATGGTTCACGTAGTGGGCCATCGCCCTGATAGACGGTTTTTCGCCCTTTGACGTTGGAGTCCACGTTCTTTAATAGTGGACTCTTGTTCCAAACTGGAACAACACTCAACCCTATCTCGGGCTATTCTTTTGATTTATAAGGGATTTTGCCGATTTCGGCCTATTGGTTAAAAAATGAGCTGATTTAACAAAAATTTAACGCGAATTTTAACAAAATATTAACGTTTACAATTTTATGGTGCACTCTCAGTACAATCTGCTCTGATGCCGCATAGTTAAGCCAGCCCCGACACCCGCCAACACCCGCTGACGCGCCCTGACGGGCTTGTCTGCTCCCGGCATCCGCTTACAGACAAGCTGTGACCGTCTCCGGGAGCTGCATGTGTCAGAGGTTTTCACCGTCATCACCGAAACGCGCGAGACGAAAGGGCCTCGTGATACGCCTATTTTTATAGGTTAATGTCATGATAATAATGGTTTCTTAGACGTCAGGTGGCACTTTTCGGGGAAATGTGCGCGGAACCCCTATTTGTTTATTTTTCTAAATACATTCAAATATGTATCCGCTCATGAGACAATAACCCTGATAAATGCTTCAATAATATTGAAAAAGGAAGAGTATGAGTATTCAACATTTCCGTGTCGCCCTTATTCCCTTTTTTGCGGCATTTTGCCTTCCTGTTTTTGCTCACCCAGAAACGCTGGTGAAAGTAAAAGATGCTGAAGATCAGTTGGGTGCACGAGTGGGTTACATCGAACTGGATCTCAACAGCGGTAAGATCCTTGAGAGTTTTCGCCCCGAAGAACGTTTTCCAATGATGAGCACTTTTAAAGTTCTGCTATGTGGCGCGGTATTATCCCGTATTGACGCCGGGCAAGAGCAACTCGGTCGCCGCATACACTATTCTCAGAATGACTTGGTTGAGTACTCACCAGTCACAGAAAAGCATCTTACGGATGGCATGACAGTAAGAGAATTATGCAGTGCTGCCATAACCATGAGTGATAACACTGCGGCCAACTTACTTCTGACAACGATCGGAGGACCGAAGGAGCTAACCGCTTTTTTGCACAACATGGGGGATCATGTAACTCGCCTTGATCGTTGGGAACCGGAGCTGAATGAAGCCATACCAAACGACGAGCGTGACACCACGATGCCTGTAGCAATGGCAACAACGTTGCGCAAACTATTAACTGGCGAACTACTTACTCTAGCTTCCCGGCAACAATTAATAGACTGGATGGAGGCGGATAAAGTTGCAGGACCACTTCTGCGCTCGGCCCTTCCGGCTGGCTGGTTTATTGCTGATAAATCTGGAGCCGGTGAGCGTGGGTCTCGCGGTATCATTGCAGCACTGGGGCCAGATGGTAAGCCCTCCCGTATCGTAGTTATCTACACGACGGGGAGTCAGGCAACTATGGATGAACGAAATAGACAGATCGCTGAGATAGGTGCCTCACTGATTAAGCATTGGTAACTGTCAGACCAAGTTTACTCATATATACTTTAGATTGATTTAAAACTTCATTTTTAATTTAAAAGGATCTAGGTGAAGATCCTTTTTGATAATCTCATGACCAAAATCCCTTAACGTGAGTTTTCGTTCCACTGAGCGTCAGACCCCGTAGAAAAGATCAAAGGATCTTCTTGAGATCCTTTTTTTCTGCGCGTAATCTGCTGCTTGCAAACAAAAAAACCACCGCTACCAGCGGTGGTTTGTTTGCCGGATCAAGAGCTACCAACTCTTTTTCCGAAGGTAACTGGCTTCAGCAGAGCGCAGATACCAAATACTGTCCTTCTAGTGTAGCCGTAGTTAGGCCACCACTTCAAGAACTCTGTAGCACCGCCTACATACCTCGCTCTGCTAATCCTGTTACCAGTGGCTGCTGCCAGTGGCGATAAGTCGTGTCTTACCGGGTTGGACTCAAGACGATAGTTACCGGATAAGGCGCAGCGGTCGGGCTGAACGGGGGGTTCGTGCACACAGCCCAGCTTGGAGCGAACGACCTACACCGAACTGAGATACCTACAGCGTGAGCTATGAGAAAGCGCCACGCTTCCCGAAGGGAGAAAGGCGGACAGGTATCCGGTAAGCGGCAGGGTCGGAACAGGAGAGCGCACGAGGGAGCTTCCAGGGGGAAACGCCTGGTATCTTTATAGTCCTGTCGGGTTTCGCCACCTCTGACTTGAGCGTCGATTTTTGTGATGCTCGTCAGGGGGGCGGAGCCTATGGAAAAACGCCAGCAACGCGGCCTTTTTACGGTTCCTGGCCTTTTGCTGGCCTTTTGCTCACATGT

*LHA*

GCGGCCGCACGCGTTTAATTAAGTGTCTAATTGGACAGGGAGATGGCTCAGGTGTGGAAAGCCAATATAGCGGTGCATGCTTGCAAGGCCAGGAGTAATGATGGGGACATAGGAGGATTCTGGGAGCCTGGCCGGATAACCTATCCCCTAACACATAAGCACCAGGTCCCACCCAGTAAGAGGCCCTGCCTCGAAGAAGAAGTGGAGGGTCCCTGAGGAGGAAGGGCATTTGGGGTTATCCTCTGGTCTCCAAATGCATCCCTCCTCCCACACAAACACACAAAGACACTTTCACTTGTATCTGATGCCTGGTTAGCCATGAGCGTAAGCTAGGATTGTGAATTCAGCCACCCTGGTACAGCAACTATTGAGTGCTTAGTGTATACTCTGGGTTCGAAACACCGAGGACAGAAATGAATAAGATGCAGTTTCTGTCCTCAGGACCACCAGAAGGAAACCAGGACGGGTGGCATCCTGTGTAGACAGACCCCGGAGTGTGTGCGTGTTGCTTAGCTAAGTGATTTTCTGGAGTGTGTTCTGGCTCCCCGGCCGTCCTCCCGAACTAGCAGACCACTCGTGCTGCCGGAGTCCTGGAGCGACAGGAGACTCAGCCTGACTTCTCTCTTAACTCTCCCTCCGCGCAGGTTCCATAGCTCAACATGGTCCCCTGGTTCCTCCTGTCTCTGCTGCTACTTGCGAGGCCTGTGCCTGGGGTGGCCTACTCTGTGTCACTCCCGGCCTCCTTCCTGGAGGATGTAGCCGGCAGCGGGGAAGCTGAGGGTTCTTCAGCCTCTTCCCCGAGCCTGCCGCCGCCTGGGACTCCAGCCTTCAGTCCCACACCGGAGAGACCCCAGCCCACAGCTCTGGACGGCCCCGTGCCACCCACCAACCTCCTGGAAGGGATCATGGATTTCTTCCGGCAGTACGTGATGCTCATCGCGGTGGTGGGCTCGCTGACCTTCCTCATCATGTTCATAGTCTGCGCCGCCCTCATCACGCGCCAGAAGCACAAGGCCACAGCCTACTACCCATCCTCGTTCCCTGAAAAGAAGTATGTGGACCAGAGAGACCGGGCTGGGGGACCCCGTACCTTCAGCGAGGTCCCTGACAGGGCACCTGACAGCCGGCATGAAGAAGGCCTGGACACCTCCCATCAGCTCCAGGCTGACATTCTGGCTGCTACCCAGAACCTCCGGTCTCCAGCTAGAGCCCTGCCAGGCAATGGGGAGGGAGCAAAGCCTGTGAAGGGTGGGTCGGAGGAGGAGGAGGAAGAGGTGCTCAGCGGTCAGGAGGAGGCCCAGGAAGCCCCAGTATGTGGGGTCACTGAAGAGAAGCTGGGGGTCCCAGAGGAGTCGGTCTCAGCAGAGGCTGAAGGGGTTCCTGCCACCAGTGAGGGCCAAGGGGAAGCAGAAGGGTCTTTCTCCTTAGCCCAGGAATCCCAGGGAGCAACTGGTCCTCCTGAAAGTCCCTGTGCCTGCAACAGAGTCTCTCCCAGTGTC

*P2A-CreERT2*

TGTGCCTGCAACAGAGTCTCTCCCAGTGTCGGAAGCGGAGCTACTAACTTCAGCCTGCTGAAGCAGGCTGGAGACGTGGAGGAGAACCCTGGACCTATGGCCAATTTACTGACCGTACACCAAAATTTGCCTGCATTACCGGTCGATGCAACGAGTGATGAGGTTCGCAAGAACCTGATGGACATGTTCAGGGATCGCCAGGCGTTTTCTGAGCATACCTGGAAAATGCTTCTGTCCGTTTGCCGGTCGTGGGCGGCATGGTGCAAGTTGAATAACCGGAAATGGTTTCCCGCAGAACCTGAAGATGTTCGCGATTATCTTCTATATCTTCAGGCGCGCGGTCTGGCAGTAAAAACTATCCAGCAACATTTGGGCCAGCTAAACATGCTTCATCGTCGGTCCGGGCTGCCACGACCAAGTGACAGCAATGCTGTTTCACTGGTTATGCGGCGGATCCGAAAAGAAAACGTTGATGCCGGTGAACGTGCAAAACAGGCTCTAGCGTTCGAACGCACTGATTTCGACCAGGTTCGTTCACTCATGGAAAATAGCGATCGCTGCCAGGATATACGTAATCTGGCATTTCTGGGGATTGCTTATAACACCCTGTTACGTATAGCCGAAATTGCCAGGATCAGGGTTAAAGATATCTCACGTACTGACGGTGGGAGAATGTTAATCCATATTGGCAGAACGAAAACGCTGGTTAGCACCGCAGGTGTAGAGAAGGCACTTAGCCTGGGGGTAACTAAACTGGTCGAGCGATGGATTTCCGTCTCTGGTGTAGCTGATGATCCGAATAACTACCTGTTTTGCCGGGTCAGAAAAAATGGTGTTGCCGCGCCATCTGCCACCAGCCAGCTATCAACTCGCGCCCTGGAAGGGATTTTTGAAGCAACTCATCGATTGATTTACGGCGCTAAGGATGACTCTGGTCAGAGATACCTGGCCTGGTCTGGACACAGTGCCCGTGTCGGAGCCGCGCGAGATATGGCCCGCGCTGGAGTTTCAATACCGGAGATCATGCAAGCTGGTGGCTGGACCAATGTAAATATTGTCATGAACTATATCCGTAACCTGGATAGTGAAACAGGGGCAATGGTGCGCCTGCTGGAAGATGGCGATCTCGAGCCATCTGCTGGAGACATGAGAGCTGCCAACCTTTGGCCAAGCCCGCTCATGATCAAACGCTCTAAGAAGAACAGCCTGGCCTTGTCCCTGACGGCCGACCAGATGGTCAGTGCCTTGTTGGATGCTGAGCCCCCCATACTCTATTCCGAGTATGATCCTACCAGACCCTTCAGTGAAGCTTCGATGATGGGCTTACTGACCAACCTGGCAGACAGGGAGCTGGTTCACATGATCAACTGGGCGAAGAGGGTGCCAGGCTTTGTGGATTTGACCCTCCATGATCAGGTCCACCTTCTAGAATGTGCCTGGCTAGAGATCCTGATGATTGGTCTCGTCTGGCGCTCCATGGAGCACCCAGTGAAGCTACTGTTTGCTCCTAACTTGCTCTTGGACAGGAACCAGGGAAAATGTGTAGAGGGCATGGTGGAGATCTTCGACATGCTGCTGGCTACATCATCTCGGTTCCGCATGATGAATCTGCAGGGAGAGGAGTTTGTGTGCCTCAAATCTATTATTTTGCTTAATTCTGGAGTGTACACATTTCTGTCCAGCACCCTGAAGTCTCTGGAAGAGAAGGACCATATCCACCGAGTCCTGGACAAGATCACAGACACTTTGATCCACCTGATGGCCAAGGCAGGCCTGACCCTGCAGCAGCAGCACCAGCGGCTGGCCCAGCTCCTCCTCATCCTCTCCCACATCAGGCACATGAGTAACAAAGGCATGGAGCATCTGTACAGCATGAAGTGCAAGAACGTGGTGCCCCTCTATGACCTGCTGCTGGAGGCGGCGGACGCCCACCGCCTACATGCGCCCACTAGCCGTGGAGGGGCATCCGTGGAGGAGACGGACCAAAGCCACTTGGCCACTGCGGGCTCTACTTCATCGCATTCCTTGCAAAAGTATTACATCACGGGGGAGGCAGAGGGTTTCCCTGCCACAGCTTGACATGCCCCAGAACTGCTGGGACCCGAATGT

*P2A-EGFP*

TGTGCCTGCAACAGAGTCTCTCCCAGTGTCGGAAGCGGAGCTACTAACTTCAGCCTGCTGAAGCAGGCTGGAGACGTGGAGGAGAACCCTGGACCTATGGTGAGCAAGGGCGAGGAGCTGTTCACCGGGGTGGTGCCCATCCTGGTCGAGCTGGACGGCGACGTAAACGGCCACAAGTTCAGCGTGTCCGGCGAGGGCGAGGGCGATGCCACCTACGGCAAGCTGACCCTGAAGTTCATCTGCACCACCGGCAAGCTGCCCGTGCCCTGGCCCACCCTCGTGACCACCCTGACCTACGGCGTGCAGTGCTTCAGCCGCTACCCCGACCACATGAAGCAGCACGACTTCTTCAAGTCCGCCATGCCCGAAGGCTACGTCCAGGAGCGCACCATCTTCTTCAAGGACGACGGCAACTACAAGACCCGCGCCGAGGTGAAGTTCGAGGGCGACACCCTGGTGAACCGCATCGAGCTGAAGGGCATCGACTTCAAGGAGGACGGCAACATCCTGGGGCACAAGCTGGAGTACAACTACAACAGCCACAACGTCTATATCATGGCCGACAAGCAGAAGAACGGCATCAAGGTGAACTTCAAGATCCGCCACAACATCGAGGACGGCAGCGTGCAGCTCGCCGACCACTACCAGCAGAACACCCCCATCGGCGACGGCCCCGTGCTGCTGCCCGACAACCACTACCTGAGCACCCAGTCCGCCCTGAGCAAAGACCCCAACGAGAAGCGCGATCACATGGTCCTGCTGGAGTTCGTGACCGCCGCCGGGATCACTCTCGGCATGGACGAGCTGTACAAGTAACATGCCCCAGAACTGCTGGGACCCGAATGT

*RHA*

CATGCCCCAGAACTGCTGGGACCCGAATGTTGGGTCCTTGAGGGTCACCTCTTTGGTCAAGAAAGGCATTCAGCTCTAACTGCTCCTTGATACCACGTGGCTTGGCCATTGCTGGTGCCAAGGCTGACCCCGAACTGGCAGAGCCGATGCCCTCTGGTGCACCCCAGGAAACATCTCCCCAAGTTCCAGCGCCCTTAATGACTCTTGCCACCCTGGGGGCTTCACCCTAACGCACCACTTCTCTGGAAGGGGAAGGCCAGACACATGCCAGTTGGGGCTGCATGAGGCAGTCCTCAGAGCAGAAGGGGACCAGGCCAGAGGCCACCTGTGACGGGGCAAACTGCATCTCGGCTGTGGAGACCAGAGGGGCTGTTAGATTTGGAAGACATCAATGACTGGGCCTGCGGCGCAGCCCGTGTCTGGTAATACCAGGGACGGCAGAGGCGTTTGCATCTTCCCATCACCTGCAATGTCGCTGTCACTCTGCCCCTGTTCAGTGGACTTGATTAGTTAGGAAACTTCTGGAAGGGGCCCCCTACTTTATATCACAGAGTTTGCCCTAGACACCCCGTGGAAACACAAACTCAAAATCAAGTGGCTTTAGGAGCCGCTGTGCCCCTCCACAAGCTGACATGGCTACTCTAAGGGTTCCTGCTGGGCTGGCTTTGCTACGCTTTCCTCAAGCTGCTTTCTTATTACCAGGATGCCTCACAGCTACAAAGTCCAATCTCACAGCACCCGCACTGGAAAATACTGTTCTCCATTTTTTTCCAAGGCACCCTGCTTTATGATTGGCTCAGAGTTGGAATATGGGATGCAGGGCGTCTGGCTCTCTCAAGTGTCAAGCAACCCTGGCTTCCGCATGTGGGGCGAGGGGAGTGAGCAGAACTCTTCTCTGTCAGTCCACGAAGTAGACTAGAAGCCAACGGGTGCCTGGGGGCAACTGACGGTCTGGATTCTGACGCCCAGCCTGGAGCAGGGGCCTGGCCCATCCTATGCTCACACAGTGGTCTGGCAGCCTGAGCCCACACAACTCCTCAGTCCTTGACAATGCTTAGGCTCTGTTCTGAGGGACTCCCCACACCTCCATTCAGGGCTCCCCGAGTCCTCAGCTCTTCGGATGTGGATATATGACACACTAGCATAATAAATCTTGATCTGGCTTGAGTCTCTCTGGATTATCTGCACAGGTCTGCAGGAATGGGGTGTTCATGGGCTGGGGGCAGCATTGGGGGGAGGGCATCAGCCAGGTCACAGAGCTGAGACTGTGTGTGGGTGCTCACAGGTGTTGGACCCACGTACCTGAGGAGTCCTGGAGGTGGTGTGTCAAACGTACAGACACTTTGCTGCCTGGCTCTGCAGCTGGTGCAGCCATGCCACAGTCACTTCTTTGTGCTGTGGGACAAAGCTGGGGCAAAGTCATGGCTGCCAATTCCCTACAGATGGGTGAGAACTGACTGAGCCTCAGGGCGATATCAAGCTTATCGATAATCAACCTCT

## Results

### Generation and genetic confirmation of Tmem119-EGFP and Tmem119-CreERT2 knock-in lines

Access to highly specific fluorescent reporter lines for imaging and Cre-driver lines for functional manipulation is critical in our quest for understanding the roles of microglia in health and disease. Previously generated lines efficiently target microglia, but also other cells of the monophagocytic system (Wieghofer and Prinz, 2016). Among a group of more recently discovered microglia-specific genes, we chose targeting the *Tmem119* gene to generate such mouse lines (Bennett et al., 2016). Specifically, we opted for a transgenic approach that harnesses the *Tmem119* locus to drive expression, while preserving endogenous *Tmem119* expression. We designed a CRISPR/Cas9 strategy to insert EGFP or CreERT2, preceded by ribosome-skipping peptide porcine teschovirus-1 polyprotein (P2A), into the stop codon of murine *Tmem119* (Fig. 1*A*). Two founder lines were established for Tmem119-EGFP and 5 for Tmem119-CreERT2.

To determine whether the donor DNA was inserted correctly, a PCR-based approach was used with primers spanning each junction. For both of the lines generated, specific 5′ and 3′ junction PCR products were obtained, whereas no bands were observed with DNA from wild-type (WT) animals (Fig. 1*B*). This indicates specific insertion of the transgenes into the *Tmem119* locus. To further examine scarless integration at the junctions, PCR products from the CreERT2 line were subject to Sanger sequencing covering the junctions. Both 5′ and 3′ sequencing traces show endogenous bases corresponding to the WT sequence in intron 1 and the 3′ UTR (Fig. 1*C*,*D*). Additional sequencing confirmed absence of mutations across the entire targeted allele in both lines. Together, these results confirm the generation of *Tmem119-EGFP* and *Tmem119-CreERT2* lines.

Although the function of endogenous *Tmem119* is currently unknown, both its temporally distinct pattern and high expression level suggest functional importance of this gene (Bennett et al., 2016). We thus designed a knock-in approach with the objective of leaving endogenous expression intact. To test whether *Tmem119* expression is actually unaffected, we quantified *Tmem119* transcript levels in the *Tmem119-EGFP* line by qPCR. Examination of *Tmem119* expression in WT and *Tmem119-EGFP*^+/−^ mice revealed that transcript levels are comparable between genotypes (Fig. 1*E*; WT median 0.9717, *n* = 4, EGFP^+/−^ median 0.9306, *n* = 6, *p* = 0.5667^a^). We further examined TMEM119 protein expression using immunofluorescence and found no difference between WT and *Tmem119-EGFP*^+/−^ knock-in mice (Fig. 1*F*,*G*; WT median 0.9835, *n* = 4, EGFP^+/−^ median 1.117, *n* = 4, *p* = 0.20^b^) Together, these data support the conclusion that *Tmem119* knock-in mice were generated with precision.

To further test whether the genetic engineering could affect the properties of microglia, we examined basic morphologic features of reconstructed immunostained microglia in WT and *Tmem119-EGFP*^+/−^ knock-in mice (Fig. 1*H*,*I*). Using this approach, we determined that the cell body area (Fig. 1*J*; WT median 50.67 μm^2^, EGFP^+/−^ median 46.82 μm^2^, *n* = 12 microglia from 4 mice per genotype, *p* = 0.3429^c^), number of processes (Fig. 1*K*; WT median 6.167, EGFP^+/−^ median 5.667, *n* = 12 microglia from 4 mice per genotype, *p* = 0.4571^d^), convex hull area (Fig. 1*L*; WT median 2413 μm^2^, EGFP^+/−^ median 1871 μm^2^, *n* = 12 microglia from 4 mice per genotype, *p* = 0.4857^e^), and total process length (Fig. 1*M*; WT median 351.2 μm, EGFP^+/−^ median 419.6 μm, *n* = 12 microglia from 4 mice per genotype, *p* = 0.3428^f^) are comparable between microglia from control and knock-in mice. These data suggest that the genetic modification does not affect basic microglial properties.

### EGFP is faithfully expressed in microglia

**Figure 2.**
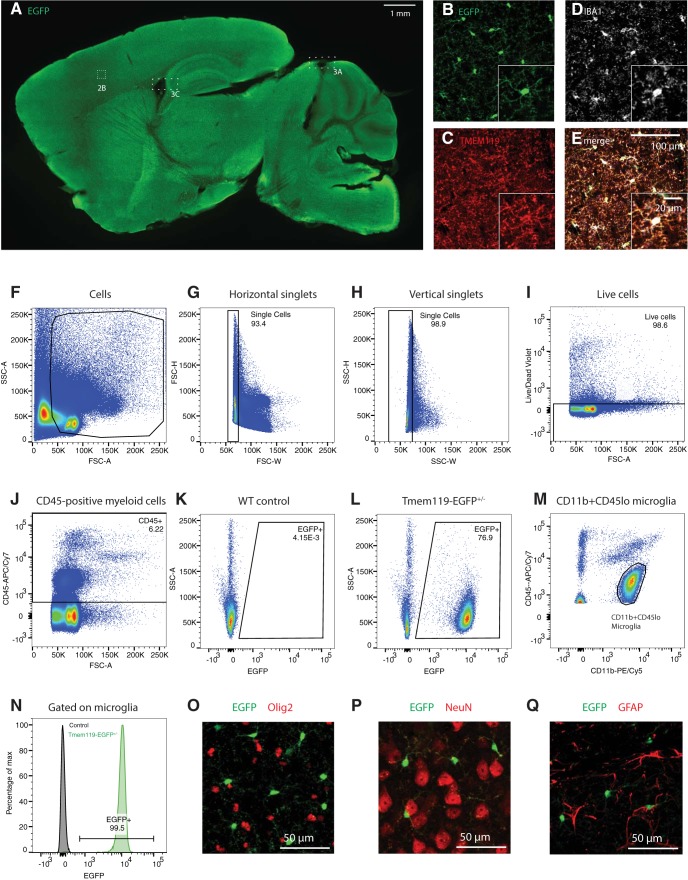
EGFP labels microglia in all regions across the brain. ***A***, Representative epifluorescence image of a sagittal section of a P25 *Tmem119-EGFP*^+/−^ mouse stained with an anti-GFP antibody for improved signal-to-noise ratio (data for 1 of 3 mice shown). ***B*–*E***, High-power confocal micrographs of native EGFP fluorescence (***B***, green) in TMEM119 (***C***, red) and IBA1 (***D***, white) microglia in the cortical region outlined in ***A***. FIJI-calculated composite image showing all three labels (***E***). Boxes 3A and 3B Boxes cross-reference areas shown in Figure 3. ***F***–***J*,** Flow cytometry analysis showing gating for of single, live, CD45-positive cells (1 of 3 independent experiments shown). Numbers in or adjacent to outlined areas indicate percentage cells in each gate. ***K***, ***L***, Representative density plots showing EGFP expression in WT and *Tmem119-EGFP*^+/−^ mice (pre-gated on CD45^+^ cells). ***M***, Representative density plot showing CD11b^+^CD45lo cells corresponding to microglia (pre-gated on CD45^+^ cells). ***N*,** Histogram showing fraction of CD11b^+^CD45lo microglia expressing EGFP. ***O***–***R***, Representative immunostaining for oligodendrocytes (***O***; Olig2, red), neurons (***P***; NeuN, red), and astrocytes (***Q***; GFAP, red) in EGFP-expressing *Tmem119-EGFP*^+/−^ mice. One of three independent experiments shown.

Microglia populate the parenchyma in virtually all regions across the brain. To determine the extent of EGFP expression in the newly generated *Tmem119-EGFP* line, we used confocal microscopy on brain slices. Native EGFP fluorescence and antibody-enhanced signal were visible across the brain in sagittal brain sections prepared from P25 *Tmem119-EGFP*^+/−^ mice (Fig. 2*A*). To further assess whether EGFP is expressed in microglia, immunofluorescence labeling of the endogenous microglia proteins IBA1 and TMEM119 was used. High-power confocal microscopy shows that EGFP-positive cells in the parenchyma are IBA1- and TMEM119-positive (Fig. 2*B*–*E*). Completion and fidelity of expression were further assessed quantitatively in several brain regions. We found that 96.1 ± 2.0% of the EGFP-expressing cells in the somatosensory cortex are IBA-1-expressing microglia and 98.2 ± 3.3% of IBA1-positive cells in this region are labeled by EGFP (Table 1). In the striatum, 99.2 ± 2.1% of the cells labeled with EGFP were IBA1-expressing and 97.2 ± 1.8% of IBA1-positive cells displayed EGFP fluorescence. Labeling of microglia in the thalamus and hippocampus was 98.5 ± 0.8% and 98.9 ± 1.9% complete and 96.9 ± 2.3% and 98.5 ± 3.5% specific, respectively. To further confirm EGFP expression in microglia across the brain, we used the *Tmem119-EGFP* line in flow cytometry (Fig. 2*F*–*N*). We gated for single, live cells (Fig. 2*F*–*I*) and detected EGFP^+^ cells among CD45^+^ cells (Fig. *J*–*M*). We determined that all CD11b^+^CD45lo microglia (Fig. 2*K*) expressed EGFP (Fig. 2*N*). In addition, to more comprehensively assess specificity of EGFP expression, we immunostained oligodendrocytes, neurons, and astrocytes. We found no evidence for expression of EGFP in these major CNS cell types (Fig. 2*O*–*Q*). Together, these data indicate highly complete and specific labeling of parenchymal microglia in the *Tmem119-EGFP* reporter line.

**Table 1.** Quantification of EGFP labeled microglia in Tmem119-EGFP mice

Brain region	Completeness, % (EGFP^+^Iba^+^/Iba^+^)	Fidelity, % (EGFP^+^Iba^+^/EGFP^+^)
Somatosensory cortex	98.2 ± 3.3	96.1 ± 2.0
Striatum	97.2 ± 1.8	99.2 ± 2.1
Thalamus	96.9 ± 2.3	98.5 ± 0.8
Hippocampus	98.5 ± 3.5	98.9 ± 1.9

**Figure 3.**
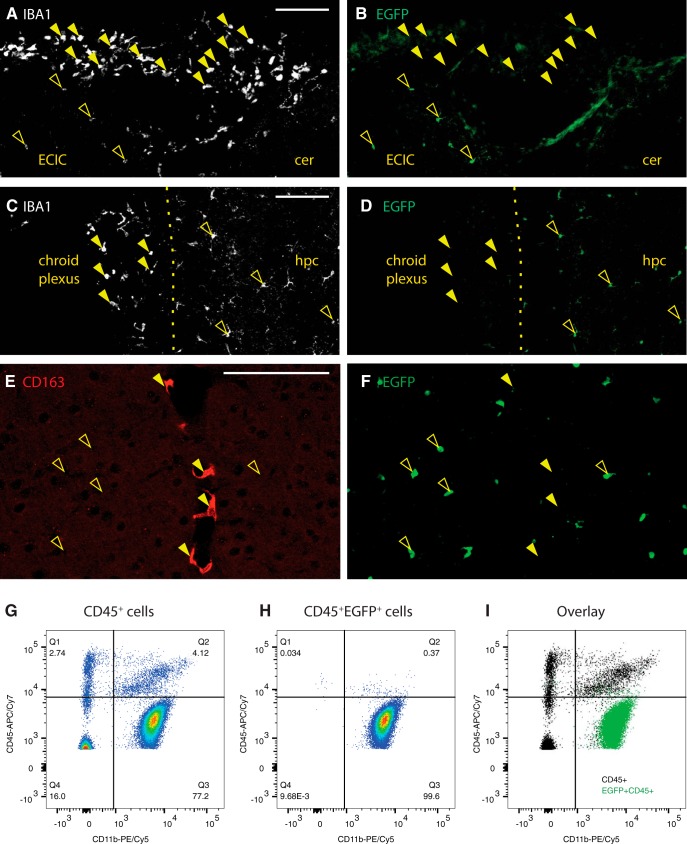
EGFP discerns parenchymal microglia from other brain macrophages in the Tmem119-EGFP line. ***A***, IBA1 labels meningeal macrophages and parenchymal microglia from the region indicate with a dashed box in Figure 2*A*. ***B***, EGFP labels only parenchymal macrophages (open arrowheads), but not meningeal IBA1-positive macrophages (closed arrowheads). Diffuse fluorescence is associated with pia. ECIC, External cortex of inferior colliculus; Cer, cerebellum. Scale bar, 100 μm. ***C***, ***D***, Choroid plexus IBA1^+^ macrophages (closed arrowheads) from the region boxed in Figure 2*A* are not labeled with EGFP, whereas adjacent parenchymal microglia are (open arrowheads). Hpc, Hippocampus. Scale bar, 100 μm. ***E***, ***F***, Perivascular CD163-expressing macrophages (***E***, red, closed arrowheads) are not labeled with EGFP (***F***, green, open arrowheads). Scale bar, 100 μm. ***G***–***I***, Flow cytometry analysis of CD11b and CD45 expression in pre-gated single live CD45^+^ cells and EGFP-expressing CD45^+^ cells of *Tmem119-EGFP*^+/−^ mice shown as density plots (***G***, CD45^+^ and ***H*** CD45^+^ EGFP^+^) and overlay (***I***, black CD45^+^, green CD45^+^ EGFP^+^). One of three independent experiments (*N* ≥ 3 mice per group) shown. Numbers in or adjacent to outlined areas indicate percentage cells in each gate.

All microglia express IBA1, but not all IBA1-expressing cells are parenchymal macrophages. In fact, non-parenchymal macrophages such as meningeal, perivascular, and choroid plexus macrophages, express the myeloid and macrophage marker IBA1 (Prinz and Priller, 2014). To determine whether EGFP is expressed in these non-parenchymal macrophages, we examined EGFP immunofluorescence in IBA1-expressing cells in the meninges and choroid plexus, which can be readily discerned by their morphology and IBA1 immunoreactivity. High-power confocal images from both regions, including adjacent parenchyma, show that, whereas parenchymal microglia express the EGFP label, neither IBA1-positive meningeal (Fig. 3*A*,*B*) nor choroid plexus macrophages express EGFP (Fig. 3*C*,*D*).

In contrast to the clear anatomic location of choroid plexus and meningeal macrophages, sites of perivascular macrophages are not easily discernible anatomically. To examine whether perivascular macrophages are labeled in the *Tmem119-EGFP* line, we stained for CD163, a well-characterized marker for perivascular macrophages (Kim et al., 2006). CD163 readily identified perivascular macrophages, which did not express EGFP (Fig. 3*E*,*F*). Adjacent microglia were clearly labeled with EGFP.

To further assess the specificity of EGFP expression in microglia beyond anatomic location-based immunofluorescence analysis, we used flow cytometry (Fig. 3*G*–*I*). Examining CD45^+^EGFP^+^ cells, we determined that 99.6% are CD11b^+^CD45lo cells corresponding to microglia (Fig. 3*H*). We further detected that 153 of 2212 CD11b^+^CD45int/hi cells (6.9%) corresponding to non-microglial cells express EGFP (Fig. 3*G*–*I*). Together, these data indicate the capacity of the *Tmem119-EGFP* line to distinguish parenchymal microglia from other brain macrophages with good selectivity.

### EGFP is expressed in microglia of Tmem119-EGFP mice at early postnatal stages

**Figure 4.**
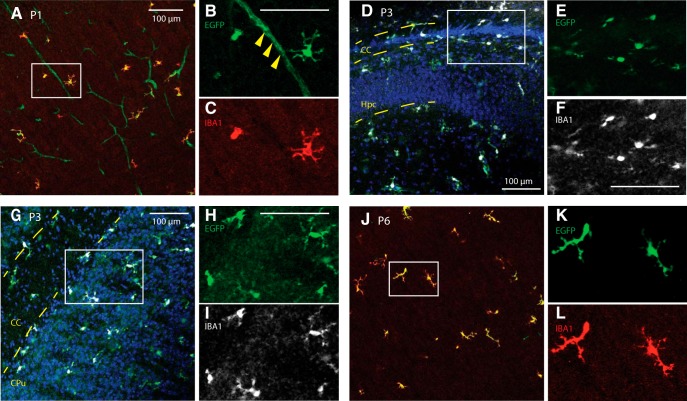
*Tmem119-EGFP* mice label microglia at early postnatal stages. Sagittal slices from P1, P3, and P6 mice were stained against IBA1. ***A***–***C***, High-power confocal images of the somatosensory cortex show EGFP expression in IBA-positive cells and blood vessels at P1 (solid arrowheads in magnified panel). ***D***–***F***, Confocal micrographs around the forceps major of corpus callosum (CC) and hippocampus (Hpc) show EGFP-labeled, IBA1-positive microglia at P3. ***G***–***I***, Confocal micrographs of the striatum (CPu) and CC show EGFP labeling of IBA-positive cells at P3. ***J***–***L***, Confocal micrographs of the P6 cortex reveal EGFP-expression in IBA-immunostained cells. Error bars, 100 μm.

Microglia populate the brain during early embryonic development (Ginhoux et al., 2010). *Tmem119* mRNA is expressed throughout microglial development, but antibody-based methods currently consistently label TMEM119 only as of later postnatal stages, typically P14 and later (Bennett et al., 2016). This makes it difficult to identify and discern microglia from monocytes at earlier time points. To determine whether the *Tmem119-EGFP* line would specifically label microglia at early postnatal stages, we conducted immunofluorescence microscopy at P1, P3, and P6. EGFP expression in IBA1-positive cells was readily observed in several regions examined, suggesting translation of *Tmem119-P2A-EGFP* transcript at this stage (Fig. 4*A*–*L*). Surprisingly, we also found EGFP expression associated with blood vessels of P1 mice (Fig. 4*A*–*C*), but not P3 or later, indicating that the transcript is also processed in cells of the intracerebral microvascular compartment at this stage. Together, these data indicate utility of the *Tmem119-EGFP* line for early developmental studies of microglia.

### Cre activity is specifically inducible in microglia of Tmem119-CreERT2 mice

**Figure 5.**
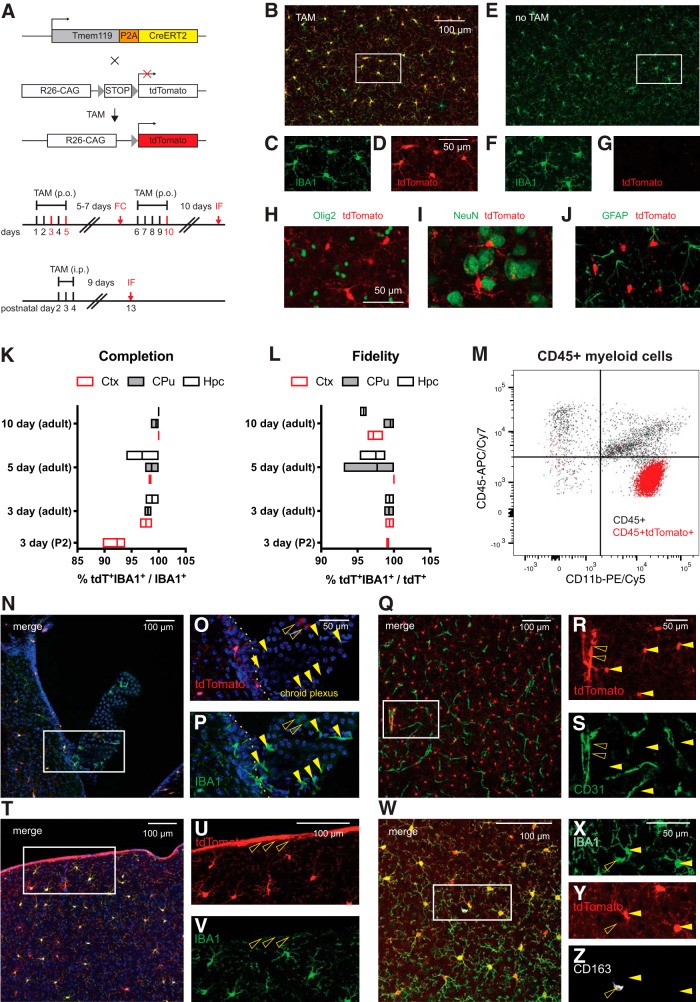
*Tmem119-CreERT2* mice effectively recombine a conditional allele in microglia. ***A***, *Tmem119-CreERT2* mice were crossed to *Ai14(RCL-tdT)-D* mice and tamoxifen (TAM) was administered to induce tdTomato expression. Different sets of adult mice received tamoxifen per os (p.o.) for 3, 5, or 10 d and a set of neonatal mice received TAM for 3 d intraperitoneally (i.p). The mice were killed for flow cytometry (FC) 7 d after the 3 d dosing paradigm or for immunofluorescence (IF) 9 or more days after dosing. ***B***–***D***, Representative confocal micrograph showing tdTomato expression in IBA-positive microglia upon tamoxifen administration. ***E***–***G***, Absence of tdTomato expression in untreated animals. ***H***–***J***, Representative immunostaining for oligodendrocytes (***H***, Olig2, green), neurons (***I***, NeuN, green), and astrocytes (***J***, GFAP, green) in tdTomato-expressing microglia of *Tmem119-CreERT2*^+/−^*; Ai14(RCL-tdT)-D*^+/−^ mice. One of three independent experiments shown. ***K***, ***L***, Percentage completion (***K***) and fidelity (***L***) of the labeling in different brain regions for the different dosing schemes. Hpc, Hippocampus; CPu, caudate–putamen; Ctx, cortex. *N* = 2 mice for 3 d, 3 mice each for 5 and 10 d of TAM in adults, and 3 mice for 3 d administration at P2. Floating bars = min to max, line at mean. ***M***, Flow cytometry analysis of CD11b and CD45 expression in pre-gated single live CD45^+^ cells and tdTomato-expressing CD45^+^ cells of *Tmem119-CreERT2*^+/−^*; Ai14(RCL-tdT)-D*^+/−^ mice shown as overlay (***I***, black CD45^+^, red CD45^+^ tdTomato^+^). One of two representative experiments (*N* = 3 mice). ***N***–***O***, Confocal micrograph of the choroid plexus and adjacent parenchyma. ***Q***–***S***, Confocal micrograph of CD31-immunostained blood vessels (green) shows tdTomato^+^ microglia (arrowheads) and tdTomato expression in a large blood vessel (open arrowhead). ***T***–***V***, Confocal micrograph showing tdTomato expression in cortical microglia and cells of the pia (open arrowheads). ***W***–***Z***, Confocal micrograph showing CD163- (white) and IBA1-expressing (green) macrophages. The open arrowhead indicates a tdTomato^-^CD163^+^IBA1^+^ perivascular macrophage. Closed arrowheads parenchymal microglia. One of three independent experiments shown (*N* = 3 mice).

Cre activity in CreERT2 fusion protein is induced by the estrogen receptor antagonist tamoxifen and can be assessed in the presence of a reporter construct. To assess whether *Tmem119-CreERT2* mice display Cre activity in parenchymal microglia on induction, we crossed *Tmem119-CreERT2* mice to the Ai14 reporter line (Madisen et al., 2010), which expresses tdTomato in the presence of Cre (Fig. 5*A*). We administered tamoxifen daily for 3, 5, or 10 d to adult mice and 3 d to P2 pups, and compared recombination efficiency and fidelity for these different paradigms (Fig. 5*A*). The mice were killed 9–10 d after the last dose and subjected to immunofluorescence staining. High-power images from confocal microscopy showed tdTomato-labeling of IBA1-expressing microglia in mice that received tamoxifen (Fig. 5*B*–*D*) and absence of tdTomato expression without tamoxifen (Fig. 5*E*–*G*). Confocal micrographs also indicated absence of tdTomato expression in other major cell types of the CNS, such as oligodendrocytes, neurons, and astrocytes (Fig. 5*H*–*J*). Assessment of the completeness of labeling as the ratio of tdTomato^+^IBA1^+^ double-positive microglia (tdT^+^IBA1^+^) to all parenchymal microglia (IBA1^+^) revealed very high efficiency of recombination (Fig. 5*K*). Similarly, we examined the fidelity of labeling which represents the fraction of actual parenchymal microglia (tdT^+^IBA1^+^) among all tdTomato-labeled cells (tdT^+^) in different parenchymal regions, and found high specificity close to 100% (Fig. 5*L*). Of note, increasing the days of tamoxifen administered to mice appeared to increase the fraction of microglia undergoing recombination. At the same time, prolonged administration of tamoxifen correlated with slightly reduced fidelity (Fig. 5*K*,*L*). We further sought to assess the specificity of tdTomato expression using flow cytometry. Among a total of 1690 CD11b^+^CD45int/hi cells in the CNS, 33 were positive for tdTomato, indicating minimal, yet nonzero, expression in non-microglial myeloid cells.

The flow cytometry data revealed that recombination was largely, but not 100% specific. Furthermore, when examining the sections, we noticed some tdTomato fluorescence that did not appear to be associated with parenchymal microglia. To explore the nature of this ectopic fluorescence in this dosing paradigm, we imaged cells in the choroid plexus (Fig. 5*N*–*P*) as well as cells in the cortex, which were immunostained for the endothelial cell marker CD31 (Fig. 5*Q*–*S*). In the choroid plexus, there was very weak tdTomato fluorescence and most IBA1-positive macrophages did not express tdTomato (Fig. 5*N*–*P*). In the cortex, high-power confocal micrographs showed that the tdTomato and the CD31-immunolabeling signal were largely separate, with some tdTomato overlap with larger CD31-positive blood vessels (Fig. 5*Q*–*S*). Furthermore, tdTomato fluorescence was present on the pia in IBA1-negative cells (Fig. 5*T*–*V*) and absent from CD163-expressing perivascular macrophages (Fig. 5*W*–*Z*). Together, these data indicate that the *Tmem119-CreERT2* line can be used to conditionally control gene expression in adult and early postnatal microglia with good specificity.

### Characterization of transgene expression in monocytes from Tmem119 knock-in mice

**Figure 6.**
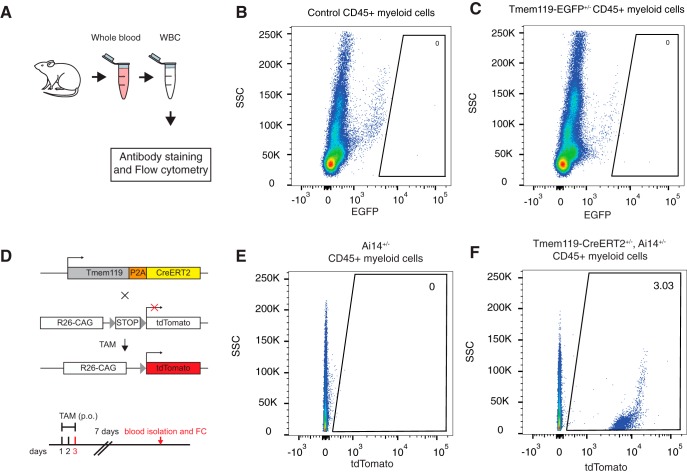
Blood monocyte profiling in Tmem119 knock-in mice. ***A***, Experimental approach for flow cytometry of monocytes isolated from whole blood. ***B***, ***C***, Representative flow cytometry analysis of EGFP expression in pre-gated single live CD45^+^ cells in WT control (***B***) and *Tmem119-EGFP*^+/−^ mice (***C***). One of three experiments is shown (*N* = 3 mice). Numbers in or adjacent to outlined areas indicate percentage cells in each gate. ***D***, Adult mice of *Tmem119-CreERT2*^+/−^*; Ai14(RCL-tdT)-D*^+/−^ mice received tamoxifen (TAM) per os (p.o.) for 3 d. The mice were sacrificed 7 d after the last dose for flow cytometry (FC). ***E***, ***F***, Representative flow cytometry analysis of tdTomato expression in pre-gated single live CD45^+^ cells in *Ai14(RCL-tdT)-D*^+/−^ (***E***) and *Tmem119-CreERT2*^+/−^*; Ai14(RCL-tdT)-D*^+/−^ mice (***F***). One of two independent experiments is shown (*N* = 3 mice).

Monocytes infiltrate the brain and contribute to pathology in a number of brain disorders. Currently widely used transgenic microglia lines also target monocytes in addition to microglia and cannot readily distinguish the two cell types, thus hampering unequivocal attribution of microglia function in these disorders. To assess the utility of *Tmem119* knock-in mice for investigating microglia in models of CNS disorders, we isolated blood monocytes and examined transgene expression (Fig. 6*A*). Using flow cytometry on white blood cells isolated from control or *Tmem119-EGFP*^+/−^ mice, we did not detect any EGFP-expression (Fig. 6*B*,*C*). To further determine whether *Tmem119-CreERT2*^+/−^*; Ai14*^+/−^ mice display recombination in monocytes, we administered tamoxifen and killed the mice for analysis 7 d after the last dose (Fig. 6*D*). Using flow cytometry on white blood cells, we found that ∼3% of CD45^+^ cells expressed tdTomato (Fig. 6*E*,*F*). This suggests that there is a low level of CreERT2 activity in monocytes of the *Tmem119-CreERT* mice. Together, these data indicate high applicability of *Tmem119-EGFP* mice and good applicability of *Tmem119-CreERT2* mice to the study of brain disorders with potential monocyte contribution.

**Table 2.** Statistical table

	**Data structure**	**Type of test**	**95% Confidence interval of difference between medians**
a	*N* too small to test normality	Mann–Whitney test	−0.3393 to 0.2262
b	*N* too small to test normality	Mann–Whitney test	−0.1601 to 0.3359
c	*N* too small to test normality	Mann–Whitney test	−11.59 to 7.300
d	*N* too small to test normality	Mann–Whitney test	−2.000 to 1.000
e	*N* too small to test normality	Mann–Whitney test	−963.9 to 560.5
f	*N* too small to test normality	Mann–Whitney test	−109.0 to 120.0

## Discussion

In this study, we addressed the critical need for transgenic mouse lines that specifically label or control microglia, but not other closely related cell types with overlapping signature gene expression (Bennett et al., 2016; Wieghofer and Prinz, 2016; Haimon et al., 2018). We targeted the recently identified microglia-specific *Tmem119* gene to knock-in *EGFP* or *CreERT2*. Multiple analyses confirm correct insertion of the transgenes, intact expression of endogenous *Tmem119*, and normal basic morphology of microglia in the knock-in mice. Using immunofluorescence and flow cytometry approaches, we demonstrate that EGFP is expressed in microglia, but not other cells of the monophagocytic system defined anatomically and by signature protein expression. Flow cytometry further shows that EGFP is almost exclusively expressed in CD11b^+^CD45lo cells corresponding to microglia. We further provide evidence for early postnatal expression of EGFP and activity of CreERT2 in microglia on tamoxifen administration. In addition, we show that blood monocytes do not express EGFP and display minimal activity of CreERT2. Together, our findings indicate proper generation of the two mouse lines, which are now readily available through a public repository (JAX#031823 for *Tmem119-EGFP* and JAX#031820 for *Tmem119-CreERT2*).

TMEM119 is a single transmembrane protein that is highly expressed in microglia in the brains of humans and mice (Bennett et al., 2016; Satoh et al., 2016). In this study, we opted to refrain from knocking *Tmem119* out through insertion of *EGFP* or *CreERT2* into the *Tmem119* ORF and instead chose to preserve expression by using a polycistronic knock-in approach with ribosomal skipping peptide P2A (Fig. 1*A*). This was primarily motivated by the high expression levels of *Tmem119*, conservation of expression in microglia across species, and potential developmental regulation patterns, all of which suggest the importance of this gene. Disruption of important genes in microglia could lead to confounding haploinsufficiency phenotypes, as previously shown for other transgenic microglia lines (Lee et al., 2010; Rogers et al., 2011). Our data rule out such potential confounding effects by showing intact *Tmem119* mRNA and protein expression (Fig. 1*E*–*G*), as well as normal basic morphology of microglia in the knock-in mice (Fig. 1*H*–*M*).

Examination of EGFP expression and co-expression analysis with TMEM119, IBA1, and CD163 revealed that EGFP labels only parenchymal microglia (Figs. 2, 3). Identification of these cells was possible by direct observation of EGFP fluorescence as well as by analysis of immunostaining-enhanced tissue sections, supporting the conclusion that EGFP expression levels are robust. Flow cytometry analysis further showed expression of EGFP in virtually all CD11b^+^CD45lo microglia (Fig. 2*N*) and a minor fraction of 6.9% (153/2212) of CD11b^+^CD45int/hi cells (Fig. 3*G*–*I*). This reflects that *Tmem119* expression is highly selective, but not completely restricted to microglia, which has been observed in recent single-cell sequencing studies (Li et al., 2019). An interesting observation was the expression of EGFP at early postnatal time points in microglia (P1, P3, and P6), but also endothelial cells of blood vessels at P1 (Fig. 4). This is interesting for two reasons. First, it suggests that *Tmem119* is initially expressed in microglia and endothelial cells and only becomes a specific microglia marker during early postnatal development, presumably by selective upregulation and downregulation of expression in microglia and endothelial cells, respectively. Second, translational processing of *Tmem119* transcript appears to stand in opposition to a previous report showing that, while RNA sequencing detects *Tmem119* mRNA throughout development, immunostaining only identifies TMEM119 protein at later postnatal stages (Bennett et al., 2016). Although our data also do not directly show TMEM119 protein expression at early stages, ribosomal processing of the transcript is at least indirectly implied by the presence of EGFP protein, suggesting TMEM119 protein might be expressed but undetected because of expression level, protein stability, differential posttranslational modification, or methodological limitations in immunostaining and flow cytometry. Together, these data indicate that *Tmem119* knock-in mice may enable the study of microglia and endothelial cells until P1 and microglia specifically after P3 and later.

Our experiments using *Tmem119-CreERT2*^+/−^ Ai14(RCL-tdT)^+/−^ mice show tdTomato expression in IBA1-expressing microglia, indicating that this line is suitable for Cre-dependent manipulation of genes and transgene expression in microglia without affecting other major neuronal or glial cell types in the CNS (Fig. 5). In this particular experiment, we observed very high, dose-dependent recombination efficiencies close to 100% with a trend for slight reduction of the generally high specificity with increasing tamoxifen dosage (Fig. 5*K*,*L*). The recombination efficiency observed across all doses compares well with other published models, such as the *Cx3cr1* mouse line (Goldmann et al., 2013), and might even permit shorter dosing paradigms dependent on the assay and relevant conditional allele. We further examined tissues other than parenchymal microglia and observed that recombination was largely yet not completely absent in choroid plexus macrophages (Fig. 5*N*–*P*). This is consistent with reported endogenous TMEM119 expression and other reports in the literature, suggesting other brain macrophages may express microglia-specific genes at very low levels (Bennett et al., 2016; Goldmann et al., 2016; Mildner et al., 2017), which might be sufficient to cause occasional all-or-none recombination events. In addition, our data showed tdTomato expression in a few larger blood vessels and IBA1-negative cells in the pia (Fig. 5*Q*–*V*). Although we did not determine the precise cellular origin of this signal, our data from CD31-immunostaining exclude endothelial cells of the intracerebral microvasculature (Fig. 5*Q*–*S*). Together with the spatial extent of tdTomato expression and recent single-cell RNA sequencing data of vascular cells, our observations point to leptomeningeal cells and their projections that penetrate deep into the brain ensheathing large blood vessels and the choroid plexus stroma (Decimo et al., 2012, Vanlandewijck et al., 2018). Together, these data render the *Tmem119-CreERT2* line a highly useful tool for the conditional study of microglia, while also suggesting potential application for leptomeningeal cells.

Examination of EGFP and tdTomato expression in blood monocytes of *Tmem119-EGFP* and *Tmem119-CreERT2; Ai14* mice revealed complete absence and minimal expression, respectively (Fig. 6). While, the lack of EGFP expression in blood monocytes of the *Tmem119-EGFP* knock-in mice was expected based on previous observations, tdTomato expression in 3% blood monocytes of the *Tmem119-CreERT2; Ai14* mice was rather surprising. Similar to other brain macrophages discussed above, blood monocytes may express *Tmem119* at very low, almost undetectable levels, which may nonetheless be sufficient to cause occasional all-or-none Cre recombination events. Together, these data render the *Tmem119* knock-in lines powerful tools to investigate microglia in health and disease without significant confounds from monocyte contribution.

During the past decade, available microglia lines have been instrumental in advancing our understanding of microglia. These lines remain powerful tools to study microglia function. At the same time, it is widely acknowledged that ontogenetic differences, as well as differences in localization and microenvironment between parenchymal microglia and their closely related cells from the monophagocytic system warrant more discerning observation and manipulation (Bennett et al., 2016; Wieghofer and Prinz, 2016; Haimon et al., 2018). Recently developed antibodies for TMEM119 immunostaining are one tool that has been adopted quickly to identify parenchymal microglia. As TMEM119 is a transmembrane protein localizing to processes, immunostaining for this protein is particularly powerful to study morphology of microglia. To identify discrete microglia, however, relatively diffuse staining patterns of membrane-bound TMEM119 present a challenge (Fig. 2*C*). In this case, the more discretely identifiable cytosolic distribution of EGFP in the *Tmem119-EGFP* line might be of great value (Fig. 2*B*). In addition, the robustness of the EGFP signal in the *Tmem119-EGFP* line may enable cell sorting without the need for antibodies, possibly even at early developmental stages before the onset of TMEM119 appearance on the membrane, as well as *in vivo* imaging approaches. Furthermore, the absence of EGFP fluorescence in blood monocytes renders this line uniquely useful for the study of microglia in brain disorders that involve monocyte infiltration. The *Tmem119-CreERT2* line will significantly facilitate conditional control of gene expression selectively in microglia, especially in cases when monocytes, but not leptomeningeal cells, could likely confound interpretations. Compared with constitutive Cre lines, which cumulatively recombine throughout development and thus deny temporal control, the *CreERT2* line will allow precise control of gene expression to examine the role of microglia in temporally restricted developmental processes. In this capacity, dose-dependency of CreERT2 and potentially resulting mosaic expression of recombined alleles using low doses may allow the study of candidate genes in adjacent microglia populations sharing the same microenvironment. Beyond the application of *Tmem119* knock-in and other currently available mouse lines, genetic approaches such as targeting other microglia-signature genes, engineering highly specific artificial promoters and regulatory regions, and using intersectional split-Cre approaches hold great promise to produce even more specific tools to study microglia in the future.

## Synthesis

Reviewing Editor: Lorna Role, NINDS Decisions are customarily a result of the Reviewing Editor and the peer reviewers coming together and discussing their recommendations until a consensus is reached. When revisions are invited, a fact-based synthesis statement explaining their decision and outlining what is needed to prepare a revision will be listed below. The following reviewer(s) agreed to reveal their identity: Jun Chen.

This is an interesting paper describing the creation of a new mouse model using the microglia-specific Tmem119 gene that would be useful for specifically labeling and manipulating microglia in the mouse brain. This is a novel and potentially important tool that the field of glial neurobiology would benefit by (all other mouse models also target other non-microglia cells).

The reviewers converged quickly on a consensus opinion on this report: Although this report is potentially of tremendous utility, the manuscript per se could be further improved by including more control experiments and more detailed information as specified below.

Overall:

The reviewers request that more details are provided about:

(a) the number of litters generated,

(b) the number of animals examined overall and samples for each assay described

(c) the sex distribution of the animals studied.

Furthermore, although the n's are mentioned for some comparisons which involved statistics, the values are small. For this mouse model to be useful, it must be reliable and establishing reliability requires seeing the same evidence multiple times.

Specifically:

1. As the authors stated, it is important to verify the impact of genetic engineering on the expression of endogenous Tmem119 itself. The reviewers request that in addition to examining the expression of Tmem119 at mRNA level (Fig. 1), that the authors also examine Tmem 119 at the protein level, e.g. by western blotting or immunofluorescence.

2. It would be helpful if the authors provide evidence on whether the genetic engineering changes the properties of microglia? For example, could the authors include in the revised version data on the basic morphological characterizations of microglia shape, volume, number of processes, etc..

3. The authors show that in the Tmem119-EGFP mouse brains, more than 96% of total Iba1+ cells were targeted, and more than 96% of EGFP+ cells express Iba1.

Adding double-label immunofluorescence of EGFP with other CNS cell markers as negative controls (e.g. neurons, astrocytes, etc.) would strengthen the specificity of the targeting approach. Similar for the Tmem119-CreER:Ai14 mice.

It is noted that these first 3 experiments should be able to be done by extraction of data from their existent set of slides.

4. The authors performed immunofluorescence to examine whether the targeting strategy would affect CNS macrophages (meningeal, choroid plexus and perivascular macrophages). These data were largely based on the anatomical location. Adding another means of characterization, for example by flow cytometry to show the exclusive expression of EGFP/Ai14 on the brain CD11b+CD45low population only, will further strengthen this claim.

5. Under various neurological disorders, the blood brain barrier is damaged and blood monocytes will infiltrate the brain. Considering the potential application of these two strains in investigating microglia under CNS disorders, it would be helpful to characterize whether blood monocytes are targeted by the Tmem119 approach.

The reviewers felt that all of the requested changes should be readily accomplished within the 2 month period required for a manuscript to be deemed as potentially acceptable, pending full re-review.

## Author Response

Detailed response to reviewers' comments:

We thank the reviewers for their support of this work and their constructive comments. As detailed below, we have revised the manuscript according to reviewers' comments by including new experimental data. We believe that the manuscript is significantly improved and we thank the reviewers for their help.

Detailed responses:

The species studied is not mentioned in the abstract. Please make sure to update both the abstract in the article file and on the submission form.

Authors' response: Thank you for bringing this up. To highlight the species better, we added “in the knock-in mouse models” in line 15.

The sex of the species studied is not mentioned in the materials and methods section. Please mention “male”, “female”, “x males and x females”, “of either sex”.

Authors' response: We appreciate that this is being pointed out, especially given recent literature of sexually dimorph features in microglia. We used mice of either sex and adjusted the manuscript accordingly in lines 86-88: “For all experiments, at least three independent mice were analyzed, which included both sexes and no apparent sex differences were observed.”

Comments on the Visual Abstract for Author (Required):

The authors may want to revisit the visual abstract as it was felt that it did not convey the scientific message of the paper.

Authors' response: Thank you very much for this comment. We examined the visual abstract again and decided to change it to better reflect the fact that these are knock-in mice. We also decided to include a piece of data in the visual abstract.

Synthesis Statement for Author (Required):

This is an interesting paper describing the creation of a new mouse model using the microglia-specific Tmem119 gene that would be useful for specifically labeling and manipulating microglia in the mouse brain. This is a novel and potentially important tool that the field of glial neurobiology would benefit by (all other mouse models also target other non-microglia cells).

The reviewers converged quickly on a consensus opinion on this report: Although this report is potentially of tremendous utility, the manuscript per se could be further improved by including more control experiments and more detailed information as specified below.

Overall:

The reviewers request that more details are provided about:

a) the number of litters generated,

Authors' response: We now included this information as follows in line 85-86: “Overall, more than 10 litters were generated per line and mice were crossed to C57BL/6J up to generation N3.”

b) the number of animals examined overall and samples for each assay described

Authors' response: Based on this request, we included the overall number of mice in line 85 “more than 30 animals of more than 10 litters” and line 87 “at least three independent mice”, as well as more detailed information in each figure legend whenever relevant.

c) the sex distribution of the animals studied

Authors' response: We did not systematically compare males and females, but used both genders equally. This is now reflected in line 86-88 “For all experiments, at least three independent mice were analyzed, which included both sexes and no apparent sex differences were observed.”.

Furthermore, although the n's are mentioned for some comparisons which involved statistics, the values are small. For this mouse model to be useful, it must be reliable and establishing reliability requires seeing the same evidence multiple times.

Specifically:

1. As the authors stated, it is important to verify the impact of genetic engineering on the expression of endogenous Tmem119 itself. The reviewers request that in addition to examining the expression of Tmem119 at mRNA level (Fig. 1), that the authors also examine Tmem 119 at the protein level, e.g. by western blotting or immunofluorescence.

Authors' response: We appreciate this concern and addressed it accordingly. We tried both western blotting and immunofluorescence staining to test for TMEM119 protein levels. For western blotting, we tried two antibodies from Abcam and five pre-release antibodies from Cell Signaling Technologies. Despite attempting several different protein preparations, we were unable to detect a credible signal corresponding to TMEM119 in control tissues, which is consistent with other groups' findings. We therefore used immunofluorescence and quantified expression in four controls and four Tmem119-EGFP+/- mice. Using this method, we found no significant difference in TMEM119 protein expression. The data have been added to Figure 1 as panels F and G.

2. It would be helpful if the authors provide evidence on whether the genetic engineering changes the properties of microglia? For example, could the authors include in the revised version data on the basic morphological characterizations of microglia shape, volume, number of processes, etc..

Authors' response: Thank you for suggesting this interesting control experiment. To address whether basic morphology of microglia is affected by the transgenic approach, we examined microglia from control and Tmem119-EGFP+/- mice. We did not find any difference in a number of basic morphological parameters such as cell body area, convex hull area, number of processes, and total process length. The data have been added to Figure 1 as panels H-M.

3. The authors show that in the Tmem119-EGFP mouse brains, more than 96% of total Iba1+ cells were targeted, and more than 96% of EGFP+ cells express Iba1.

Adding double-label immunofluorescence of EGFP with other CNS cell markers as negative controls (e.g. neurons, astrocytes, etc.) would strengthen the specificity of the targeting approach. Similar for the Tmem119-CreER:Ai14 mice.

Authors' response: We agree that it is relevant to address whether any neurons, oligodendrocytes or astrocytes are targeted by the transgenic approach. As suggested, we tested this using immunofluorescence labeling of oligodendrocytes with Olig2, neurons with NeuN, and astrocytes with GFAP in both lines of transgenic mice. We did not find any expression of either EGFP or tdTomato in any of these other cell types. The data have been added to figure 2 panels O-Q for the EGFP line and figure 5 panels H-J for the CreERT2 line.

4. The authors performed immunofluorescence to examine whether the targeting strategy would affect CNS macrophages (meningeal, choroid plexus and perivascular macrophages). These data were largely based on the anatomical location. Adding another means of characterization, for example by flow cytometry to show the exclusive expression of EGFP/Ai14 on the brain CD11b+CD45low population only, will further strengthen this claim.

Authors' response: Thank you for the excellent insight. We agree with the referees' point that additional characterization with a second experimental method would be tremendously useful. Using flow cytometry, we characterized transgene expression in CD11b+CD45+ population in both transgenic lines. The data from these experiments support the claim that EGFP and CreERT2 are selectively expressed in CD11b+CD45lo cells. We did also find some cells (2-7%) of the CD11b+CD45int/hi population that expressed the transgenes. These data have been added to figure 3 as panels G-I and figure 5 as panel M. As these data do indicate some amount of expression in these other myeloid populations, we felt that it would be appropriate to slightly adjust a number of statements to reflect this.

These statements now read:

Lines 36-39 in significance statement: “Complementing and significantly extending recently introduced microglia-specific immunostaining methods that quickly became a new standard in the field, we generated two mouse lines that label and control gene expression in microglia with high specificity and made them publicly available.”

Lines 73-74 in introduction: “We demonstrate that EGFP is expressed throughout the brain and that the tag is confined to microglia only, without significantly labeling other brain macrophages.”

Lines 556-558 in results: “Together, these data indicate the capacity of the Tmem119-EGFP line to distinguish parenchymal microglia from other brain macrophages with good selectivity.”

Lines 595-597 in results: “Among a total of 1690 CD11b+CD45int/hi cells in the CNS, 33 were positive for tdTomato, indicating minimal, yet nonzero, expression in non-microglial myeloid cells.”

5. Under various neurological disorders, the blood-brain barrier is damaged and blood monocytes will infiltrate the brain. Considering the potential application of these two strains in investigating microglia under CNS disorders, it would be helpful to characterize whether blood monocytes are targeted by the Tmem119 approach.

Authors' response: Thank you very much for suggesting this great experiment. Using flow cytometry, we characterized blood monocytes in both lines of mice. We found that blood monocytes in the Tmem119-EGFP line do not express EGFP. In the Tmem119-CreERT2 line, we observed tdTomato expression in 3% of CD45+ myeloid cells. We feel that this adds to the manuscript, as it indicates excellent utility of the Tmem119-EGFP line and decent utility of the Tmem119-CreERT2 line for the study of brain disorders with potential monocyte infiltration. These data have been included as figure 6.
